# A comparative study of the phenotype with kainic acid-induced seizure in DBA/2 mice from three different sources

**DOI:** 10.1186/s42826-020-00072-y

**Published:** 2020-10-28

**Authors:** Kyung-Ku Kang, Young-In Kim, Min-Soo Seo, Soo-Eun Sung, Joo-Hee Choi, Sijoon Lee, Young-Suk Jung, Joon Young Cho, Dae Youn Hwang, Sang-Joon Park, Kil Soo Kim

**Affiliations:** 1 Laboratory Animal Center, Daegu-Gyeongbuk Medical Innovation Foundation, Daegu, Republic of Korea; 2 KPC Corporation, Gwangju, 12773 Korea; 3 College of Pharmacy, Pusan National University, Busan, 46241 Korea; 4 Exercise Biochemistry Laboratory, Korea National Sport University, 88-15 Oryung-dong, Songpa-gu, Seoul, 138-763 Korea; 5 Department of Biomaterials Science, College of Natural Resources & Life Science/Life and Industry Convergence Research Institute, Pusan National University, Miryang, 50463 Korea; 6 Department of Histology, College of Veterinary Medicine, Kyungpook National University, Daegu, 702-701 Korea; 7 College of Veterinary Medicine, Kyungpook National University, 80 Daehakro, Buk-gu, Daegu, 41566 Korea

**Keywords:** DBA/2Kor1, Kainic acid, Seizure, Brain, DBA/2 mouse

## Abstract

The kainic acid-induced seizure mouse model is widely used in epilepsy research. In this study, we applied kainic acid to the subcutaneous injections of three different sources of DBA/2 mice to compare and evaluate the seizure response. The three mouse sources consisted of DBA/2Kor1 (Korea FDA source), DBA/2A (USA source), and DBA/2 (Japan source), and were purchased from different vendors. To compare the responses of DBA/2 mice to kainic acid injections, we examined the survival rate, seizure phenotype scoring, and behavioral changes. We also evaluated brain lesions using histopathological analysis. Following the administration of kainic acid, almost half of the cohort survived, and the seizure phenotype displayed a moderate level of sensitivity (2 ~ 4 out of 6). In the histopathologic analysis, there was no change in morphological features, and levels of glial fibrillary acidic protein (GFAP) and ionized calcium binding adaptor molecule 1 (Iba-1) increased in the kainic acid-treated groups. However, there was no difference in the neuronal nuclei (NeuN) expression level. All the data showed that the responses in the kainic acid-treated group were similar across the three strains. In conclusion, our results suggest that the three sources of DBA/2 mice (DBA/2Kor1, DBA/2A, and DBA/2B) have similar pathological responses to kainic acid-induced seizures.

## Introduction

Epilepsy is a major nervous system disease with a worldwide distribution. Approximately 50 cases of epilepsy incidence per 100,000 people in the general population occur annually, and the lifetime probability of seizures is approximately 3% [1]. Several mechanisms for epilepsy have been identified. Among them, oxidative stress has been identified as the main mechanism of seizure activity. Inflammation also plays an essential role in epilepsy development [2].

Administration of the excitatory toxic substance kainic acid (KA) can stimulate the glutamate receptor and is commonly used to model seizures in rodents. The administration of KA causes the upregulation of reactive oxygen species (ROS) and glutamine activity [3]. Increasing evidence has revealed that oxidative stress is a potential molecular mechanism of KA-induced neurotoxicity and is associated with hippocampal cell death [4].

Outbred mice are widely used in biomedical research and industry. DBA/2 mice were established at the National Institutes of Health [5]. Rodents, including DBA/2 mice, have generally been used as animal models for a long time. KA-induced seizure studies are performed using DBA/2 mice [6–9] and include toxicity, brain and biological response tests [10–14]. Among laboratory animals, DBA/2 mouse is a commonly used model.

In this study, we compared the effect of KA subcutaneous injection in DBA/2 mice obtained from three different sources (DBA/2Kor1, DBA/2A; U.S. origin and DBA/2B; Japan origin). In addition, DBA/2Kor1 mice established by the Korean FDA were evaluated responses and phenotypes. Our results are the first evidence to show a significantly similar response to KA-induced seizures in DBA/2Kor1, DBA/2A and DBA/2B mice.

## Methods/experimental

### Animals

Male DBA/2 mice (six-week-old) were obtained from three separate sources. The DBA/2 mice were purchased from vendors located in Korea (DBA/2Kor1), the United States (DBA/2A), and Japan (DBA/2B). The experimental animal protocols were reviewed and approved by the Institutional Animal Care and Use Committee of KPC Co. Ltd. Kyunggido, Korea (KPC-IACUC; approval No. P191114) and were in accordance with their guidelines. All mice were given ad libitum access to a standard irradiated chow diet (Purina, Seoul, Korea) and sterilized water. During the study period, the mice were maintained in specific pathogen-free conditions under a strict light cycle (light on at 7:00 and off at 19:00, 12-h dark-light cycle), 23 ± 2 °C, and 50 ± 10% relative humidity at the KPC animal facility.

### Seizure induction and phenotype scoring

**Fig. 1:**
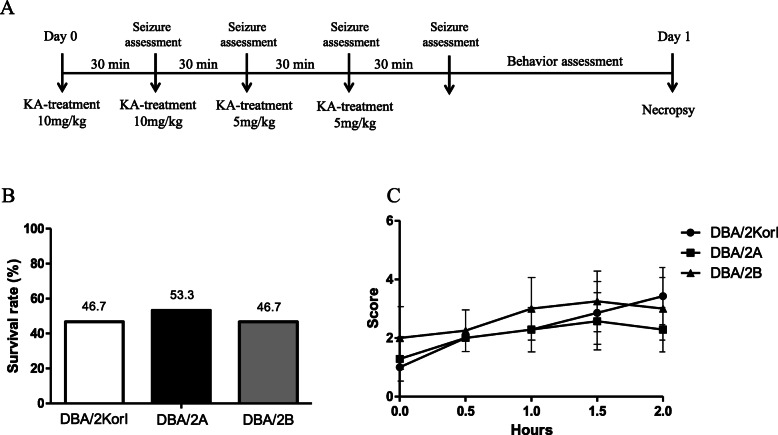
Scheme of KA-induced seizure model in DBA/2 mice (**a**). Survival rate after one day after KA injections (**b**, *n* = 15/group) and seizure phenotype scoring (**c**, *n* = 7/group) among the three strains. The data represent the mean ± SD

The three types of mice (DBA/2Kor1, DBA/2A, and DBA/2B) were each divided into two groups (negative control group and kainic acid-induced group; *n* = 15 per group). Prior to the experiment, all mice were acclimatized for 7 days. Kainic acid monohydrate (K0250, Sigma-Aldrich, St. Louis, MO, USA) was injected into the kainic acid-induced group mice, and saline was injected into the negative control group mice. The kainic acid was dissolved in 0.1 M phosphate-buffered saline at a concentration of 5 mg / mL and was freshly prepared on the day of each experiment. The mice were separated into individual cages and given subcutaneous kainic acid injections; the initial dose was 10 mg/kg. After 30 min, the mice were evaluated according to an established six-point seizure scale, as described in a previous study (Table 1) [15]. In the experimental group, we observed whether the seizures were induced by repeated applications at 30-min intervals for 2 h following administration by subcutaneous injection with a final dose of 30 mg/kg (10, 10, 5, and 5 mg/kg) for the kainic acid treatment group (Fig. 1a). The survival rate was measured after the seizure observation for 1 day was completed, excluding dead mice.

**Table 1:** Established six-point seizure scale

Level	Characteristic behaviors
1	Unmoving and crouched in a corner, staring
2	Body stretched out, tail becomes straight and rigid, ears laid back, bulging eyes
3	Repetitive head bobbing, rears into a sitting position with forepaws resting on belly
4	Rearing and falling, tonic-clonic seizures broken by periods of total stillness, jumping clonus, running clonus
5	Continuous level 4 seizures
6	Body in clonus, no longer using limbs to maintain posture, usually precursor to death

### Y-maze test

Short-term spatial recognition memory performance was assessed by recording spontaneous alternation behavior in a single Y-maze session. After the seizure observation concluded, each mouse was placed at the end of one arm and allowed to move freely through the maze during an 8-min session. The series of arm entries was visually recorded. Alternation was defined as successive entries into the three arms on overlapping triplet sets. The maximum number of spontaneous alternations was then calculated as the total number of arms entered minus two, and the percentage was calculated as the ratio of actual to potential alternations.

### Histopathological analysis

Following the experiment, all mice were anesthetized in a chamber using 2% isoflurane (Virbac, UK). Brain tissues were extracted for histopathology analysis and fixed with 10% neutral buffered formalin solution (BBC Biochemical, Mount Vernon, WA, USA) for 1 week. The fixed tissues were embedded in a paraffin block and sectioned into 4-μm thick sections. The sections were then mounted on slides and stained with hematoxylin and eosin (H&E) solution using an autostainer (Dako Coverstainner; Agilent, Santa Clara, CA, USA). Additionally, the tissue sections were stained immunohistochemically using the labeled polymer DAKO EnVisionTM+System-HRP (Agilent) according to the manufacturer’s instructions. The brain sections were stained with anti-GFAP (ab7260; Abcam, Cambridge, MA, USA), anti-Iba1 (ab5076; Abcam, Cambridge, MA, USA), and anti-NeuN (ab177487; Abcam, Cambridge, MA, USA) primary antibody. After staining, all areas of the brain that were scanned with a slide scanner (Pannoramic SCAN II; 3DHISTECH, Budapest, Hungary) and were captured by a slide viewer (CaseViewer; 3DHISTECH, Budapest, Hungary). For quantification analysis, Each slide captures 3 points at 400x in the hippocampus. Using image analyzer software Image J (NIH, MD, USA), the positive portion of the total is quantified as a percentage and the average is calculated.

### Statistical analysis

The data were analyzed using one-way ANOVA (Graphpad Prism; Graphpad, CA, USA) for identify significant differences among groups and were represented as mean ± standard error in graphical plots. Statistically significant differences are indicated with asterisks (*p < 0.05).

## Results

### Survival rate and seizure phenotype scoring

For comparative evaluation of seizure behavior in DBA/2 mice using kainic acid, the survival rate of the three DBA/2 mouse strains was measured 1 day after kainic acid administration. As a result, it was confirmed that the survival rates between DBA/2Korl, DBA/2A, and DBA/2B mice showed similar trends: 46.7, 53.3, and 46.7%, respectively (Fig. 1b). The level of seizure behavior following the administration of kainic acid was divided into six stages, as described in previous research [15]. The seizure score was determined through observation of the behaviors associated with corresponding levels. The higher the score, the more severe the seizure is. It was confirmed that seizures with a score of 2 to 4 were induced within 2 h approximately 30 min after kainic acid administration in the three strains, and there was no significant difference in any strain (Fig. 1c). Therefore, as a result of seizure level evaluation, it was confirmed that the trends were similar across the three groups.

### Y-maze test

**Fig. 2:**
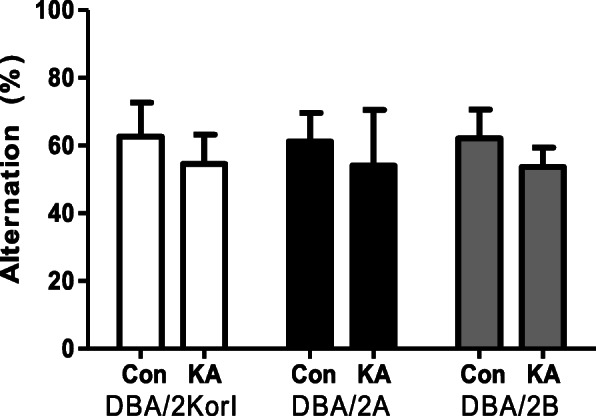
Y-maze alteration of the three strains of DBA/2 mice following KA injections (*n* = 7/group). The data represent the mean ± SD

The Y-maze test was performed as a behavioral evaluation experiment for seizure behavior following the administration of kainic acid for the three DBA/2 mouse strains. In the Y-maze results, the control and kainic acid groups were compared to the three strains (DBA/2Korl, DBA/2A, and DBA/2B). As a result, Y-maze alternation behavior was not significant in the group that was administered kainic acid, however decreasing alternation was confirmed (DBA/2Kor1 Control: 62.7%, KA: 54.6%; DBA/2A Control: 61.2%, KA: 54.1%; DBA/2B Control: 62.1%, KA: 53.6%) (Fig. 2). It was confirmed that these behavioral results showed similar trends in the three mouse groups (DBA/2Kor1, DBA/2A, and DBA/2B).

### Histopathology analysis of brain

**Fig. 3:**
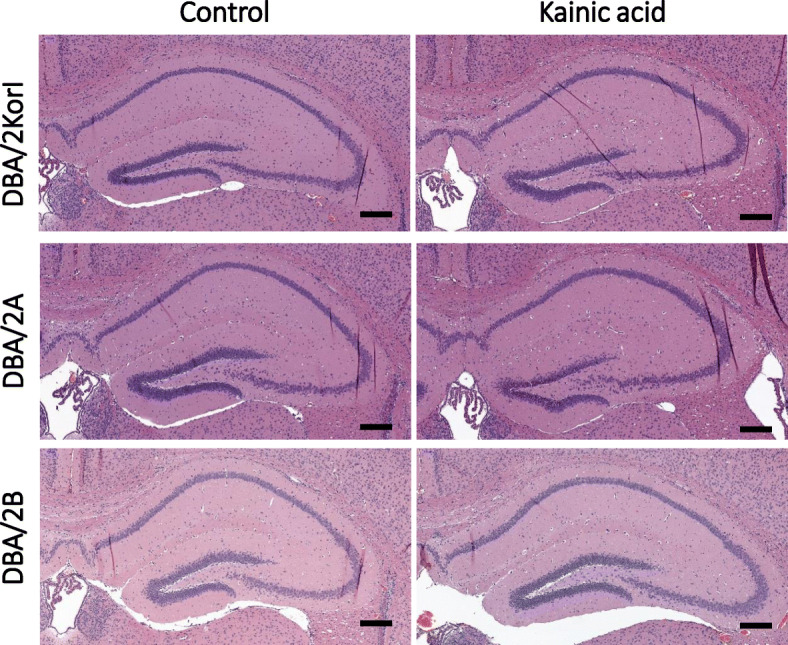
Histopathology analysis of the brain following KA-induced seizure in DBA/2 mice from three different sources (*n* = 7/group). H&E staining of the mouse brain. Scale bar = 200 μm

**Fig. 4:**
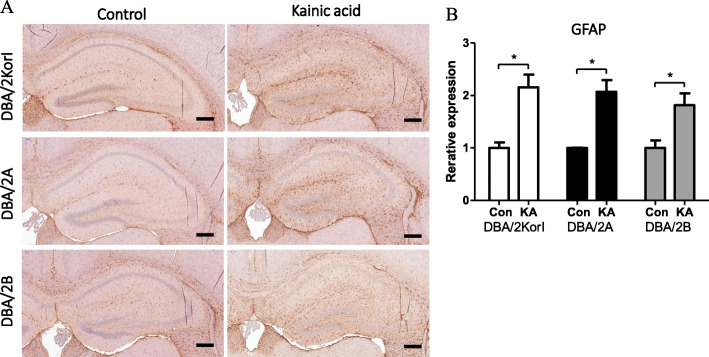
GFAP immunohistopathology analysis of the brain following KA-induced seizure in DBA/2 mice from three different sources (*n* = 7/group). GFAP-IHC staining of the mouse brain (**a**) and semi-quantitative expression data (**b**). Scale bar = 200 μm. Significantly different compared to the negative control group (*p < 0.05). The data represent the mean ± SD

**Fig. 5:**
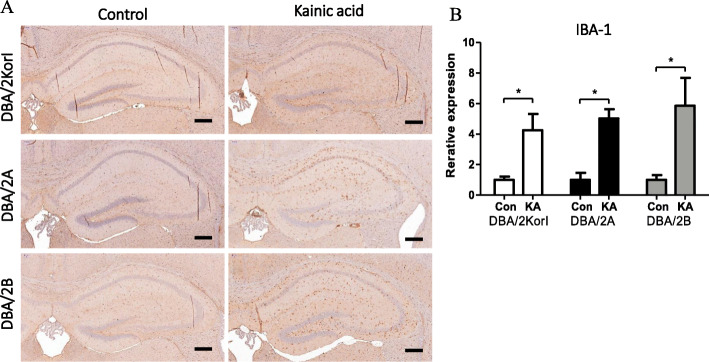
Iba-1 immunohistopathology analysis of the brain following KA-induced seizure in DBA/2 mice from three different sources (*n* = 7/group). Iba-1-IHC staining of the mouse brain (**a**) and semi-quantitative expression data (**b**). Scale bar = 200 μm. Significantly different compared to the negative control group (*p < 0.05). The data represent the mean ± SD

**Fig. 6:**
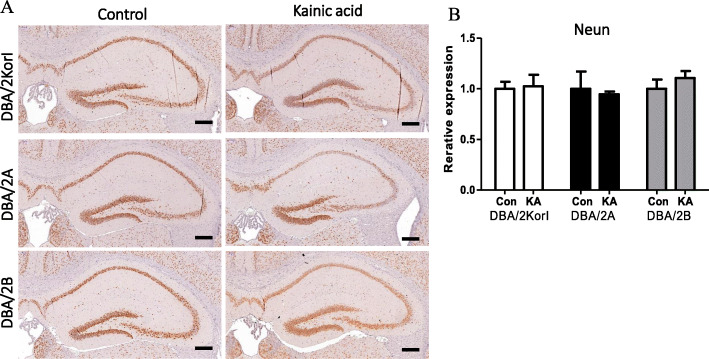
NeuN immunohistopathology analysis of brain following KA-induced seizure in DBA/2 mice from three different sources (*n* = 7/group). NeuN-IHC staining of the mouse brain (**a**) and semi-quantitative expression data (**b**). Scale bar = 200 μm. The data represent the mean ± SD

Histopathological examination was performed using H&E staining and immunohistochemistry. In the H&E staining, no significant histopathological findings were observed in the control group or the kainic acid groups of DBA/2Korl, DBA/2A, and DBA/2B mice (Fig. 3). Through immunohistochemistry, the expression of glial fibrillary acidic protein (GFAP), ionized calcium binding adaptor molecule 1 (Iba-1), and neuronal nuclei (NeuN) in the hippocampus of DBA/2 mice was confirmed. As a result of observing the mouse brain, the expressions of GFAP and Iba-1 increased in the kainic acid administration group compared to the control groups of the three mouse strains (Figs. 4, 5). However, there was no significant difference in NeuN expression (Fig. 6).

## Discussion

Studies utilizing laboratory rodents has long been carried out and applied to research in order to develop treatments for human diseases and pathophysiologies. Diverse mechanisms have been uncovered with laboratory animal models, including those as a result of toxicity and drug efficacy studies. DBA/2 mice have been previously used in KA-induced seizure studies [7–9]. However, no comparative studies have been conducted regarding DBA/2 mice from separate sources. This study investigated the response to KA-induced seizures using DBA/2Kor1, DBA/2A, and DBA/2B mice from three different sources.

We assessed seizures through histopathological analysis following KA administration as well as various physiological phenotypes, including survival rates, seizure scores, and behavioral changes in DBA/2 mice. The survival rate after KA administration in all treatment groups was close to half (46.7 ~ 53.3%). Thus, the survival rate may have occurred in the KA-treated groups due to acute disturbances, including neurodegenerative processes that are triggered in the brain [16].

To determine the seizure phenotype, we evaluated the seizure scoring system, as described in a previous study [15]. This scoring system consisted of six levels (Table 1) via checked characteristic behaviors. The results of the seizure scores confirmed that immediately after KA administration, the score was between 1 and 2 points but increased over time to become a score between 2 and 4 points after 2 h, and there was no significant difference between the three strains. The severe seizure phenotype (5 points or more) did not appear to display serious seizure characteristics with a score of 5 or higher in living mice since the severely advanced individuals had already died.

The Y-maze test was performed to evaluate the behavioral effects of the KA-induced seizure model. The Y-maze test assesses short-term spatial cognitive memory in rodents [17]. Since many neuronal pathological changes occur in the KA-induced seizure model, there have been previous studies that examined the effects of behavior, including memory [18]. The three strains were not significant but showed a similar tendency to decrease, so there was no difference in susceptibility among strains.

Inflammation is one of the hallmarks of epilepsy [19, 20]. Inflammation is consequent to acute seizure, while a lasting inflammatory response is thought to contribute to epileptogenesis [21, 22]. GFAP and Iba-1 are markers for microglia and astrocytes, which are activated during the inflammation process of the CNS. Studies using the KA model also found that cytokines are involved in microglia, and astrocyte interactions and manipulation of pre-inflammatory and anti-inflammatory cytokines can modify results concerning seizure activity, behavioral changes, and neuropathological lesions [23–26]. In this experiment, there were no histomorphological changes as a result of the administration of KA. However, it was shown that microglia and astrocyte activity was shown as inflammation markers, and inflammation and seizure caused by KA were well induced. We noted similar responses in all three strains, and GFAP and Iba-1 increased in the KA-administered group compared to the control. On the other hand, NeuN showed no change as a result of KA administration. NeuN is a neuron cell marker, and these results suggest that KA did not induce an effect on necrosis directly on the neuron.

## Conclusions

In conclusion, we tested the survival rate, seizure phenotype, and behavioral and histopathological responses to subcutaneous KA injections of DBA/2 mice originating from three separate sources. The three groups of mice (DBA/2Kor1, DBA/2A, and DBA/2B) displayed similar seizure responses to KA injections. Our results suggest that DBA/2Kor1 mice could be as widely applied to seizure studies that use KA as other DBA/2 mice that are currently commercially available.

## Data Availability

All data generated or analyzed during this study are included in this article. These data are available on request from the corresponding author.

## Abbreviations

GFAP: Glial fibrillary acidic protein
H&E: Hematoxylin and eosin
Iba-1: Ionized calcium binding adaptor molecule 1
KA: Kainic acid
NeuN: Neuronal nuclei
NLAR: National Laboratory Animal Resources
ROS: Reactive oxygen species
